# Are IgM-enriched immunoglobulins an effective adjuvant in septic VLBW infants?

**DOI:** 10.1186/1824-7288-39-63

**Published:** 2013-10-07

**Authors:** Letizia Capasso, Angela Carla Borrelli, Claudia Parrella, Silvia Lama, Teresa Ferrara, Clara Coppola, Maria Rosaria Catania, Vita Dora Iula, Francesco Raimondi

**Affiliations:** 1 Neonatal Intensive Care Unit, Department of Pediatrics, Università degli Studi di Napoli Federico II, Via S. Pansini 5, 80131 Naples, Italy; 2 Department of Cellular and Molecular Biology and Pathology, Università degli Studi di Napoli Federico II, Naples, Italy

**Keywords:** Neonate, Immunoglobulins, Sepsis

## Abstract

**Aim:**

To investigate the effectiveness of IgM-enriched immunoglobulins (IgM-eIVIG) in reducing short-term mortality of neonates with proven late-onset sepsis.

**Methods:**

All VLBW infants from January 2008 to December 2012 with positive blood culture beyond 72 hours of life were enrolled in a retrospective cohort study. Newborns born after June 2010 were treated with IgM-eIVIG, 250 mg/kg/day iv for three days in addition to standard antibiotic regimen and compared to an historical cohort born before June 2010, receiving antimicrobial regimen alone. Short-term mortality (i.e. death within 7 and 21 days from treatment) was the primary outcome. Secondary outcomes were: total mortality, intraventricular hemorrhage, necrotizing enterocolitis, periventricular leukomalacia, bronchopulmonary dysplasia at discharge.

**Results:**

79 neonates (40 cases) were enrolled. No difference in birth weight, gestational age or SNAP II score (disease severity score) were found. Significantly reduced short-term mortality was found in treated infants (22% vs 46%; p = 0.005) considering all microbial aetiologies and the subgroup affected by Candida spp. Secondary outcomes were not different between groups.

**Conclusion:**

This hypothesis-generator study shows that IgM-eIVIG is an effective adjuvant therapy in VLBW infants with proven sepsis. Randomized controlled trials are warranted to confirm this pilot observation.

## Introduction

An immature innate immune response is a major factor in the high rate of systemic infections among very low birth weight newborns. Preterm babies have less endogenous immunoglobulins whose transplacental transfer mainly occurs after 32 weeks of gestation [1-4]. Intravenous immunoglobulins supplementation is therefore an appealing strategy to fight neonatal sepsis.

A recent international, randomised trial, INIS study, on standard immunoglobulins (S-IVIG) added to antibiotic therapy in neonates with suspected infection concluded that S-IVIG had no effect on death or major disability at the age of 2 years [5]. The results of INIS study also constitute the database of a recent Cochrane review on the use of Ig for sepsis in neonate [6]. Yet, the outcome of neonatal sepsis might benefit from different IVIG preparations. Natural IgM antibodies play an important role in clearing pathogens, enhancing immune responses, and preventing autoimmunity, thus Ig-M enriched immunoglobulins (IgM-eIVIG) may have a strong therapeutic potential. In adult septic patients, the use of IgM-eIVIG as an adjuvant to antibiotic therapy has led to a significant reduction in disease severity or mortality rate in the Intensive Care Units [7,8]. There are limited data on passive immunotherapy with IgM-eIVIG in septic neonates and no specific report is focused on VLBW babies though they have the highest risk of invasive infection. We have then conducted a retrospective, cohort study on the use of IgM-eIVIG in addition to antibiotic therapy in VLBW neonates with late onset sepsis as an hypothesis generator for future prospective clinical trials.

## Methods

The charts of consecutive VLBW infants included in the local section of the Vermont Oxford Network (VON) database from January 2008 to December 2012 for a total of 491 neonates were reviewed . Neonates were born at the Università “Federico II” di Napoli, the largest delivery place in the Naples regional area assisted by a level III NICU. Inclusion criterium was the diagnosis of blood culture-proven late onset sepsis (i.e. sepsis occurring after 72 hours of life) in VLBW infants.

For defining blood culture as positive, we adopted the Vermont Oxford Network criteria, i.e.:

– sepsis by coagulase negative staphylococcus: pathogen recovered from either a central line, or peripheral blood sample in association to one or more signs of generalized infection and treatment with 5 or more days of intravenous antibiotics after the above cultures were obtained;

– sepsis by other bacteria: bacterial pathogen recovered from blood culture;

– sepsis by fungi: fungus recovered from a blood culture obtained from either a central line or peripheral blood sample [9].

Clinical signs for the diagnosis of generalized infection were: apnoea, mottled skin, temperature instability, feeding intolerance, significant abdominal distension, respiratory distress or hemodynamic instability. Laboratory criteria used were elevated CRP (cut off =1 mg/dL), abnormal leukocyte count (cut off less than 5.000/μl or more than 20.000/μl) and I/T ratio (cut off >0.2). To assess the clinical severity of cases and controls at enrollment, we used the SNAP II score, a composite index of six physical parameters (hypotension, hypothermia, acidosis, PO_2_/FiO_2_ ratio, multiple seizures, urinary output) initially designed for NICU admission [10]. A recent study validated the SNAP II score as a accurate mortality predictor at the onset of severe neonatal sepsis [11]. For this study, SNAP II score was calculated for each patients using parameters reported in the charts in the first 24 hours sepsis was suspected (i.e. when clinical deterioration was reported, blood work was done and antibiotics were started).

Exclusion criteria were: congenital anomalies, TORCH group infections, primary immunodeficit.

Intravenous antibacterial therapy used throughout the study was teicoplanin (loading dose 16 mg/Kg followed by 8 mg/Kg q 24 hours) and meropenem (20 mg/Kg q 8 hours).

In the event of a positive blood culture for Candida spp., antibiotic therapy was withdrawn and a standard antifungal regimen (liposomal amphotericin B: 5 mg/kg/die) was used for 3 weeks.

Starting from June 1st 2010, IgM-eIVIG (Pentaglobin® Biotest Germany) 250 mg/kg/day i.v. for three days was added to the NICU protocol for sepsis treatment in the first 24 hours sepsis was suspected when antibiotic therapy was started. In order to compare neonates before and after the introduction of IgM-eIVIG in our clinical practice, meticulous care was exerted to ascertain that no other major change had been made that could have been relevant to the study outcome (i.e. central lines managing and duration; fluconazole prophylaxis against invasive fungal infections; handwashing and other general prophylactic measures; management of enteral nutrition).

VLBW preterms with late onset sepsis may have a prolonged hospital stay after a septic episode and their demise might be related to many factors other than infection; therefore, short-term mortality (i.e. death within 7 and 21 days from treatment) was an appropriate primary outcome for this study. Secondary outcome measures were: in-hospital total mortality, rates of intraventricular hemorrhage, periventricular leukomalacia, necrotizing enterocolitis, bronchopulmonary dysplasia at discharge.

### Statistical analysis

ANOVA was used to compare the main characteristics of study population between treated and untreated neonates with IgM-eIVIG; to evaluate the strength of association or non –indipendence between two binary data values, odds ratios with confidence interval were calculated with SPSS 19.0 software (IBM Corporation, NY); p ≤ 0.05 was considered statistically significant.

## Results

**Figure 1:**
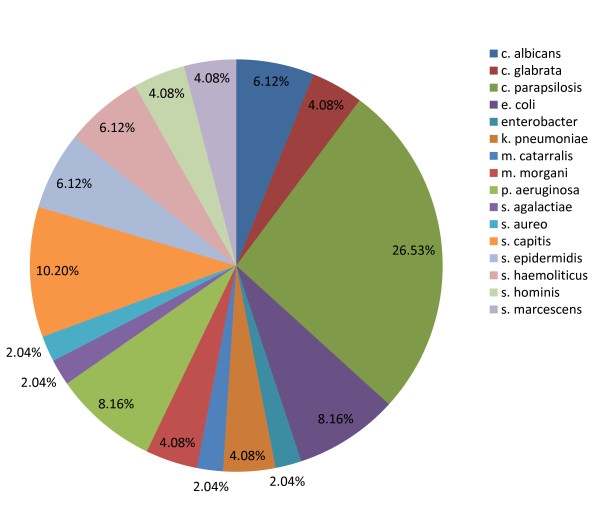
Main pathogens isolated from blood cultures.

Of the 82 VLBW infants enrolled, 2 neonates were excluded for congenital anomalies and one for TORCH infection; of the remaining 79 (see demographics in Table 1), 40 patients received antibiotics in association to IgM-eIVIG and 39 received antibiotic treatment alone. No difference in birth weight, gestational age or SNAP II score was found. All enrolled infants had a positive blood culture and their microbiology is given in Figure 1.

**Table 1:** Main characteristics of the whole study population

**IgM-eIVIG**	**Treated**	**Untreated**	
	**n = 40**	**n = 39**	**p**
**Gestational age (weeks)**	27 ± 2,6	27,6 ± 3,9	NS
**Birth weight (grams)**	924 ± 277	951 ± 362	NS
**Snap II score**	15 ± 13	12 ± 9	NS
**Cesarean section**	39 (59%)	38 (58%)	NS
**Prenatal steroids**	32 (48%)	39 (60%)	NS
**CRP positive**	51 (77%)	49 (75%)	NS

Mortality at 7 and 21 days coincided. IgM-eIVIG treated infants had a significantly lower short term mortality than untreated (OR 0.16; 95% CI: 0.3-0.7<, p = 0,005). Secondary outcomes were not significantly different between cases and controls (Table 2).

**Table 2:** Primary and secondary outcomes of the whole study population

**IgM-eIVIG**	**Treated**	**Untreated**		
	**n = 40**	**n = 39**	**OR (95%CI)**	**p**
**Short term mortality**	9 (22%)	18 (46%)	**0.16 (0.3-0.7)**	0,005*
**Total mortality**	17 (44%)	18 (46%)	0.46 (0.9-1.8)	NS
**IVH**	9 (22%)	8 (20%)	1.17 (0.5-2.7)	NS
**PVL**	3 (7,5%)	2 (3%)	2.6 (0.5-14)	NS
**NEC**	2 (6%)	1 (3%)	2 (0.4-11)	NS
**BDP**	4(10%)	4 (10%)	0.97(0.2-4.2)	NS

*p < 0.05 (IVH: intraventricular hemorrhage, PVL: periventricular leukomalacia, NEC: necrotizing enterocolitis, BPD: bronchopulmonary dysplasia).

In a subgroup analysis of neonates with Candida spp. sepsis, fewer short term deaths were also found among treated neonates. In fact 1/10 (10%) neonates died in treated group versus 8/15 (53%) deaths in untreated group (OR 0.1; 95% CI: 0.01-0.97, p = 0.047).

An accurate review of patients’ charts did not show acute reactions compatible with IgM-eIVIG infusion.

## Discussion

This pilot retrospective study shows that IgM-eIVIG are effective in reducing short-term mortality in VLBW infants with proven sepsis. When compared with S-IVIG, IgM-eIVIG show more efficient complement activation, better opsonisation, greater neutralization of the streptococcal superantigen SpeA and better binding both to bacterial antigens and toxins all possibly due to their pentameric structure [12]. This biological background together with our retrospective observation may increase the interest towards this strategy for IgM-eIVIG in VLBW infants with sepsis despite the paucity and somewhat conflicting specific clinical evidence in the literature.

In a randomized trial of 60 Saudi septic neonates of various gestational ages, Haque showed that IgM-eIVIG significantly decreased total mortality [13]. In a separate paper, the same author showed on a cohort of 195 neonates that IgM-eIVIG but not S-IVIG were effective in decreasing mortality [14]. These reports do not clearly indicate their general sepsis rate prevalence, making their results hard to compare. A negative conclusion was drawn in the study by Erdem et al. working, though, with a likely underpowered sample cohort [15].

With a larger population we detect a clinically relevant difference focusing only on the most susceptible babies. Under the standard conditions of VON, we show an effect of IgM-eIVIG on short term mortality on those babies who had culture proven sepsis.

Unlike the Haque series, our most prevalent pathogens were fungi against which IgM-eIVIG proved to be very effective. Over the study period, our incidence of Candida spp. positive blood cultures was above the Italian average but mostly below the third quartile when compared to the local section of the VON database. We later found out that the IgM-eIVIG used had an average anti C. albicans specific titer of 1.618 U/ml of IgG, 171 U/ml of IgA, 194 U/ml of IgM (courtesy of the manifacturer). The explanation for this novel observation may be related to the dominant protective role of antibodies against disseminated candidiasis. In fact, antibody opsonization is crucial to optimal Candida phagocyting and killing by neutrophils and monocytes. Moreover, antibodies against Candida mannan antigens activate complement [16].

Our retrospective study was focused strictly on short-term mortality on the assumption that a therapeutic advantage of immunotherapy would be easier to demonstrate. Total mortality is influenced by many variables other than sepsis acting long after the resolution of an infection in a ELBW or VLBW infant. It is not surprising, therefore, that short term and total mortality may differ and that secondary outcomes were not significantly different between cases and controls.

Despite the encouraging results, our study finds the main limitation in its observational nature; however, searching for confounders, we were not able to detect significant change of NICU practices or outcome variations in the study period. Also, mortality is an immediate clear-cut endpoint.

## Conclusion

This hypothesis-generator study shows that IgM-eIVIG is an effective adjuvant therapy in VLBW infants with proven sepsis reducing short term mortality.

We believe that this analysis fulfilled its original purpose to set the ground for larger, randomized prospective trials. Given the considerable burden of morbidity and mortality imposed by neonatal sepsis, new research should urgently be addressed not only to validate our results but also to tailor the optimal scheme of treatment.

## Competing interests

The authors declared that they have no competing interests.

## Authors’ contribution

LC and FR conceived the study and wrote the protocol. LC supervised data collection. FR and ACB performed statistical analysis. ACB, CP, SL, CC collected data. MRC and VDI performed microbiological analysis. LC, FR and ACB wrote the manuscript. All authors read and approved the final manuscript.
